# Circulating MicroRNAs as Specific Biomarkers in Atrial Fibrillation: A Meta-Analysis

**DOI:** 10.3390/ncrna9010013

**Published:** 2023-02-09

**Authors:** Antônio da Silva Menezes Junior, Lara Cristina Ferreira, Laura Júlia Valentim Barbosa, Daniela de Melo e Silva, Vera Aparecida Saddi, Antonio Márcio Teodoro Cordeiro Silva

**Affiliations:** 1 Internal Medicine Department, Cardiology and Genetic Section, Pontifical Catholic University of Goiás, Goiânia 74175-120, GO, Brazil; 2 Internal Medicine Department, Medicine Faculty and Biological Sciences Institute at Federal University of Goiás, Goiânia 74690-900, GO, Brazil

**Keywords:** meta-analysis, atrial fibrillation, molecular biomarkers, prognosis, diagnosis, microRNA

## Abstract

Atrial fibrillation (AF) is the most frequently occurring supraventricular arrhythmia. Although microRNAs (miRNAs) have been associated with AF pathogenesis, standard protocols for quantifying and selecting specific miRNAs for clinical use as biomarkers should be optimized. In this study, we evaluated the clinical application of miRNAs as biomarkers for the prognosis and diagnosis of AF. Literature searches were conducted on PubMed, Cochrane Library, and EMBASE. We included prospective or retrospective observational studies that had been published as of 14 February 2022; our main objective was to analyze the relationship between circulating miRNAs and AF. The data were extracted using the descriptors “Atrial fibrillation AND miRNA”, “Atrial fibrillation AND diagnostic AND miRNA”, and “Atrial fibrillation AND prognosis AND miRNA”. No filters were applied for period delimitation, type of publication, or language. Studies using samples isolated from blood plasma and TaqMan and RT-qPCR for detecting and quantifying miRNAs were selected, and those that used atrial tissue samples were excluded. We identified 272 articles and excluded 102 duplicated articles. Two authors independently read the titles and abstracts of 170 out of 272 articles and selected 56 potential articles, 6 of which were selected for final review. Our analysis revealed a significant association between AF and miR-4798 [OR = 1.90 (95% CI 1.45–2.47)], AF and miRNA-133a [2.77 (2.73–2.82)], AF and miRNA-150 [3.77 (1.50–9.46); I^2^ = 70%], AF and miRNA-21 [2.23 (1.20–4.17); I^2^ = 99%], AF and hsa-miRNA4443 [2.32 (2.20–2.44)], and AF and miR-20a-5p [3.67 (1.42–9.49)]. The association between miRNAs and AF showed an OR of 2.51 [95% CI 1.99–3.16; I^2^ = 99%]. Our meta-analysis demonstrated that circulating miRNAs are potential biomarkers of AF, as they exhibit stable expression post–sample collection. In addition to regulating cellular processes, such as proliferation, differentiation, development, and cell death, miRNAs were found to be linked to arrhythmia development.

## 1. Background

Atrial fibrillation (AF) is the most frequently occurring supraventricular arrhythmia and is associated with high morbidity and mortality rates, substantially increasing the risk of heart failure, stroke, and systemic thromboembolism [1]. AF affects ~1% of the world’s population, and its prevalence is higher in the elderly population (over 80 years of age), affecting ~8% of this population [2].

The two main pathophysiological mechanisms involved in AF development are re-entry and ectopic activity [1]. These result from changes in atrial tissue structure and function, whether induced by AF itself or by other diseases [3,4,5,6]. Such atrial remodeling leads to electrical and structural changes caused by fibrillation [7]. Atrial remodeling begins with electrical modification due to changes in action potentials and is expressed by atrial refractoriness and slower conduction time [8]. Other factors that influence the formation of arrhythmias by abnormal electrical conduction are linked to structural remodeling, which can occur by inflammatory processes, cellular hypertrophy, fibrosis, and atrial dilation [2].

AF presents numerous associated risk factors, such as hypertension, cardiomyopathies, obstructive sleep apnea, valve dysfunction, obesity, and sexual dysfunction [3]. In addition to age, family history is also considered an essential factor in the development of AF, as it presents associated genetic factors, such as autosomal dominant inheritance, in which epigenetic mechanisms can contribute to its pathophysiology through structural and functional alterations of proteins responsible for various cellular activities [4,5]. Among the genetic factors involved, microRNAs (miRNAs) play a relevant role, as they participate in electrical and structural remodeling in the evolution of AF [2].

miRNAs are small noncoding RNAs encoded by nuclear DNA and transcribed by RNA polymerase II. Their primary function is to regulate post-transcriptional gene expression by binding to complementary target sequences within the messenger RNA (mRNA), inhibiting translation or degradation of the transcribed target [2]. The biosynthesis of miRNAs begins with the activity of RNA polymerase II, which transcribes primary miRs (pri-miRs) and is subsequently recognized by the DiGeorge syndrome critical region 8 (DGCR8) protein associated with the DROSHA enzyme, forming a DROSHA–DGCR8 complex that allows the cleavage of pri-miRs originating from the precursor miRs (pre-miRs). These pre-miRs are exported to the cytoplasm by exportin-5 and cleaved by DICER, generating double-tape miRs recognized by Argonaut proteins, which in turn promotes the release of mature miRs. In association with mature miR and other proteins, the Argonaut proteins constitute the RNA-induced silencing complex, which targets specific mRNAs. After binding to mRNAs, GW182/TNRC6 proteins interact with Argonaut proteins and recruit the CCR4-NOT deadenylase complex, which induces the degradation or silencing of mRNAs [9,10,11].

F 21 miRNAs can mix with microparticles and are frequently bound to high-density proteins and lipoproteins, which protects them against RNase activity, thereby maintaining their stability in plasma and still allowing for high specificity and sensitivity [12]. Therefore, F 21 miRNAs present great potential as biomarkers of specific disease pathologies based on their particular expression in tissues. In AF, miRNAs are involved in structural and electrical remodeling processes and play essential roles in signaling during AF pathogenesis. Thus, changes in miRNA levels are related to the signaling cascade of atrial fibrosis and electrical remodeling. Therefore, different miRNAs and their quantifications may indicate variable clinical conditions and act as positive or negative markers for the development of AF [13,14]. Specific miRNAs, then, might have the potential to serve as biomarkers for screening and prediagnosis, as risk factors for AF, and in therapy, as they might interfere with the decrease in susceptibility to AF [6].

The quantification processes of specific miRNAs are faced with several challenging factors such as dosage method, gender, age, and associated comorbidities. Therefore, it is essential to develop a standard protocol for the quantification and selection of specific miRNAs to facilitate their clinical application to determine the correlation between changes in specific miRNAs and disease conditions. We performed a meta-analysis of studies reporting the associations between miRNAs and AF pathogenesis in an effort to identify the most promising AF biomarkers.

## 2. Methods

This meta-analysis followed the PRISMA 2020 protocol and is registered on the PROSPERO platform (Registry CRD42021260109).

### 2.1. Inclusion Criteria

We included prospective or retrospective observational studies published before 14 February 2022, in which the main objective was to analyze the relationship between circulating miRNAs and AF. Studies were considered eligible for the present review if they met the following criterion: patients with AF and blood plasma were used as the study sample.

### 2.2. Exclusion Criteria

Studies that did not include miRNA analysis; animal studies; reviews; pharmacological studies; studies with incomplete statistical data; studies that did not present control case analysis; studies with patients with comorbidities associated with AF such as neoplasms, depression, and stroke; and studies with patients with other cardiovascular involvements were excluded.

### 2.3. Literature Search Strategy

The literature searches were performed on PubMed, the Cochrane Library, and EMBASE using the descriptors “Atrial fibrillation AND miRNA AND biomarkers”, “Atrial fibrillation AND diagnostic AND miRNA”, and “Atrial fibrillation AND prognosis AND miRNA”, and no filters were applied for period delimitation, type of publication, or language. Two of the contributing authors (LCF and LJVB) of the present study conducted the literature search and perused all the selected papers.

### 2.4. Data Extraction and Result Definition

The following information was extracted from the selected studies: (1) details of the publication (surname of the first author and year of publication), (2) study design and study period, (3) characteristics of the studied population (sample size, sex, age, and associated comorbidities), and (4) evaluation of the result (specific relationship of the proposed miRNA with AF).

### 2.5. Statistical Analyses

To obtain the results, the effect size test was applied to combine the odds ratio (OR) and hazard ratio (HR) effect estimates. The effect size test has the potential to combine central creditors and their variances. In the present study, both effect estimates were considered: OR, which assesses a possible association between dependent and independent variables; and HR, which assesses the risk of unaffected participants to present the event. The exponential transformation was considered in the analysis; thus, it was possible to estimate how many times the study participants presented chances or risks of developing the outcome. The random effect was considered according to the I² test developed by Higgins and Thompson [15], which should be applied when the heterogeneity estimated by the I2 test has a result equal to or greater than 50%. The chi-square and Tau² tests were also considered for originating from significance of heterogeneity, considering the limit of 5%. To qualify the heterogeneity, the methodological data of the selected studies were tabulated; later, graphic preparation and analysis were performed, considering the bias summary and the bias graph. The statistical analysis was performed using Stata 16.0 [16], and the qualitative bias experiment was performed using RevMan 5.4.1 (Cochrane Collaboration, 2020).

## 3. Results

**Figure 1:**
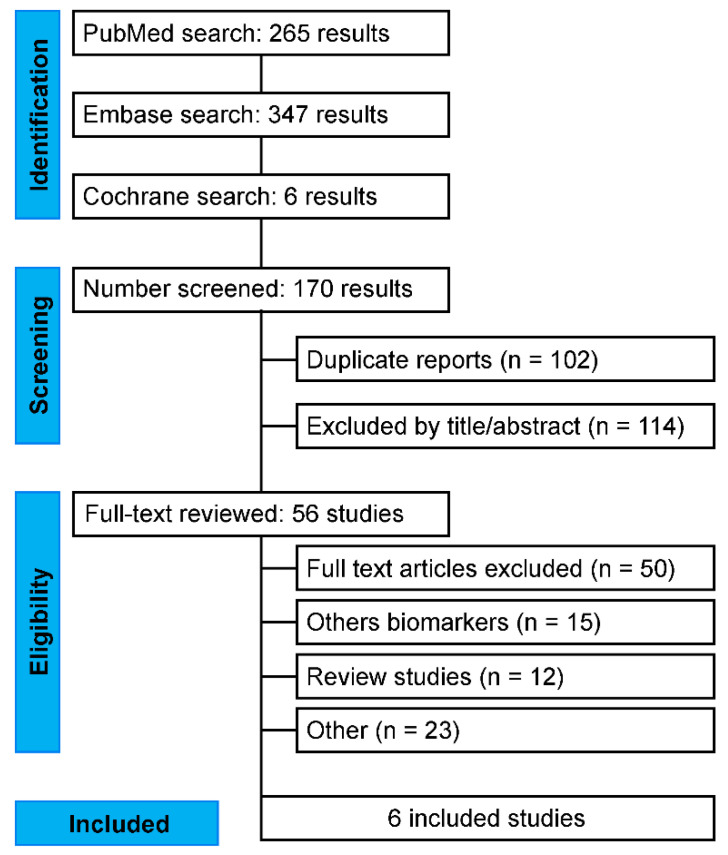
Flowchart showing the selection, inclusion, and exclusion of articles on circulating microRNAs as specific biomarkers in atrial fibrillation used for the meta-analysis.

By applying the proposed methodology of our study, we conducted the last search on 14 February 2022 on all three platforms, yielding 272 articles, of which 102 duplicates were excluded through Zotero 6.0. (Corporation for Digital Scholarship, 2022). Two authors independently read the remaining 170 articles. After reading the abstracts, the authors included the studies that met the inclusion criteria. Reviews and papers without case analyses and controls were excluded. Subsequently, two authors (L.J.V.B. and L.C.F.) read the abstracts of the selected articles and selected 56 articles on the basis of the inclusion criteria to be read in full by the mediator reviewer (A.d.S.M.J.). After complete reading, six articles were selected for this study. These studies were performed on patients with AF and control groups associated with specific plasma miRNAs as positive or negative predictors of AF. To eliminate selection bias, we included studies using samples isolated from blood plasma and those using a specific methodology for detecting and quantifying similar miRNAs, such as TaqMan and RT-qPCR, and excluded those using atrial tissue samples (Figure 1).

### 3.1. Characteristics of Selected Studies

In the study by Galenko et al. [17], which longitudinally followed the electronic medical records of an integrated intermountain health system, two populations, 140 cases of AF, 50 controls without AF, and a separate set of 141 cases of AF that had a radiofrequency catheter ablation were followed to determine the recurrence of AF over one year. The miRNA profile of the plasma samples was analyzed using quantitative TaqMan assays. 

In the case group, 50% of the patients were male and the mean age was 63.3 years. In the control group, 44% were male and the mean age was 57.9 years. Multivariate logistic regression determined the association between miRNAs and the comparison groups, and a logistic regression model was constructed for each miRNA [17]. The authors found substantially increased miRNA-21 in patients with AF. Although miRNA-21 was not associated with any AF subtypes (paroxysmal or persistent), there is evidence that miRNA-21 and its target protein are linked to the atrial fibrosis process, acting on profibrotic regulatory mechanisms such as in the transforming growth factor-beta (TGF-β)-induced pathway. However, defining miRNA-21 expression as tissue-specific is not possible because of the ambiguity about whether its variation in patients with AF is due to its expression in the atria. In addition, as shown in Table 1, the authors found an association between increased expression of miRNA-133a and a 2% increase in the chance of developing AF [17,18,19].

In a study by Liu et al. [18], the authors selected a sample of 90 participants, comprising 30 control individuals (mean age, 37 ± 13 years), 30 individuals with paroxysmal AF (mean age, 42.5 ± 13.5 years), and 30 individuals with permanent AF (mean age, 62 ± 13 years). The miRNAs in their blood plasma samples were quantified using TaqMan and miRNA reverse transcriptase-polymerase chain reaction (qRT-PCR) [20]. Patients with AF showed decreased expression levels of miRNA-146a, miR-150, miRNA-375, and miRNA-19a. In addition, the authors found remarkably reduced miRNA-150 expression in the patients with AF. That study showed a correlation between the expression of genes related to inflammatory response mechanisms, acting on the ion channels of calcium, apoptosis, and fibrosis, and the pathogenesis of AF and indicated its use as a predictor of AF (OR 1.96, 95% CI 1.5–3.57, *p* < 0.001). Additionally, compared to the control group, patients with persistent AF showed an increase in the expression of miRNA-21, which plays an essential role in regulating fibrotic remodeling.

Sieweke et al. [18], through a prospective semiblind study, investigated 81 patients, including 13 young people without AF or stroke, 10 older adults without AF or stroke, 37 patients with acute ischemic stroke, 11 patients with paroxysmal AF without stroke, and 10 patients with stroke and AF of recent onset. Plasma and serum samples were collected, and miRNAs were amplified using qRT-PCR and specific TaqMan assays. miR-21, miR-29a, miR-146b, and miR-328 were significantly decreased in patients with AF compared to the patients without AF. Further, Sieweke et al. [18] performed a multivariate regression analysis that highlighted miR-21 [HR 0.16 (95% CI 0.04–0.7), *p* = 0.009] as a predictor of the presence of AF and concluded that a decrease in miR-21 levels correlated with the presence of AF.

In a study by Xiao et al. [21], 123 patients with persistent AF (for at least six months) and 100 healthy individuals (control group) who underwent routine laboratory tests, electrocardiogram, and physical examination were selected. Among the patients with AF, 55% were men with a mean age of 61 years. In the control group, 52% were men with a mean age of 61 years. From the samples of patients and donors used as controls, qualitative and quantitative analyses of miRNAs were performed using an Agilent Bioanalyzer 2100 (Small RNA Analysis Chip; Agilent Technologies, Inc., Santa Clara, CA, USA), followed by an analysis using a TaqMan kit. This study showed that a decrease in the serum levels of the hsa-miR-4443 mRNA was associated with the pathogenesis of AF, as its expression was substantially lower in patients with AF than that in the control group. This is because hsa-miR-4443 acts as a mediator of proliferation, migration, and invasion of human cardiac fibroblasts, the most abundant cells in the heart. In addition, Xiao et al. [21] suggested that hsa-miR-4443 plays a direct role in the expression of THBS1 to activate the TGF-β pathway because its overexpression induces cardiac fibrosis, which alters the myocardial structure to produce a favorable medium for the development of AF. Thus, this study concluded that hsa-miR-4443 showed high sensitivity and specificity in diagnosing AF as an essential and promising biomarker for predicting the development of AF. However, this study was limited by its sample size.

Chen et al. [22] selected 60 patients with persistent AF and a control group of 60 individuals with corresponding sex, age, and concomitant diseases, 40% of whom were male with a mean age of 62 years. Blood samples were collected from patients of both groups, and qualitative and quantitative analyses were performed using a TaqMan kit. It was observed that the circulating concentrations of miR-21 were substantially higher in the patients with AF; this was directly linked to myocardial fibrosis levels, which are considered a predictor for the incidence of AF, with high sensitivity and specificity. Even after adjustment for variables, myocardial fibrosis levels proved to be an independent contributing factor to the development of AF. Thus, this study concluded that miR-21 could be used as a biomarker, alone or in conjunction with other clinical, echocardiographic, or serological markers, to determine the risk profile of AF. However, the expression of miR-21 in other organs could be a limitation on its use as an AF-specific marker.

Geurts et al. [19] selected 1901 participants with incident AF, of whom 42.1% were men and 57.9% were women. Compared to the men, the women were older and non-smokers and exhibited more cases of hypertension and diabetes mellitus. Among the selected patients, 98 (4.9%) had prevalent AF; of these, 6.8% were men and 3.5% were women. That study reported that the plasma levels of miRNA miR-4798-3p and miR-20a-5p were strongly associated with AF prevalence, especially in the male population, owing to the different target genes of this miRNA and its potential effects; however, its role in the pathology of AF is not yet fully clarified. Nevertheless, it is known that miR-20a-5p and other miRNAs participate in all biological pathways through the regulation of gene expression. One of the limitations of that study was the lack of differentiation between cases with paroxysmal, persistent, and longstanding AF owing to the unavailability of a 24-h Ambulatory ECG Monitoring Test in the units, as seen in Table 2.

### 3.2. Meta-Analysis Results

**Figure 2:**
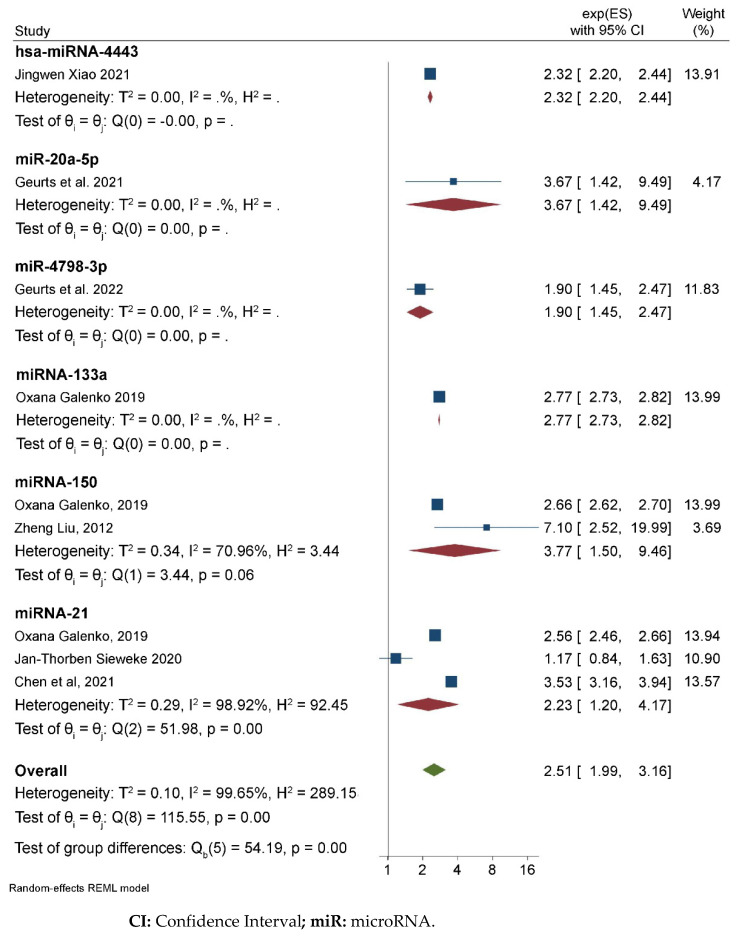
Association between miRNAs and AF.

Six articles were selected on the basis of the statistical methodology applied and estimates of the effects (Table 3). On the basis of the results from these studies, there were significant associations between AF and miR-4798 [OR = 1.90, 95% CI (1.45–2.47)]. AF and miRNA-133a [OR = 2.77, 95% CI (2.73–2.82)], AF and miRNA-150 [OR = 3.77, 95% CI (1.50–9.46); I^2^ = 70%], AF and miRNA-21 [OR = 2.23, 95% CI (1.20–4.17); I^2^ = 99%], AF and hsa-miRNA4443 [OR = 2.32 (2.20–2.44)], and AF and miR-20a-5p [OR = 3.67 (1.42–9.49)]. In general, the association between miRNAs and AF showed an OR of 2.51, 95% CI [1.99–3.16; I^2^ = 99%] (Figure 2; Table 3).

### 3.3. Evaluation of Bias

**Figure 3:**
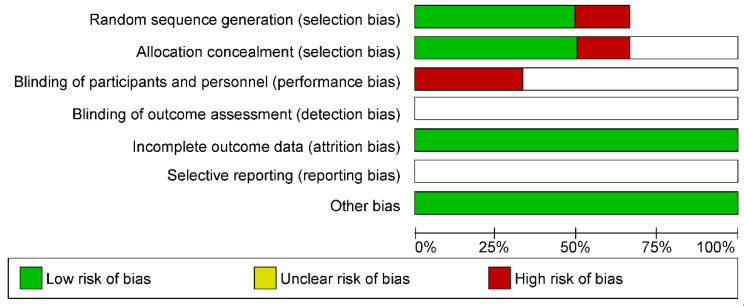
Risk of bias graph: review of authors’ judgments about each risk of bias item presented as percentages across all included studies.

**Figure 4:**
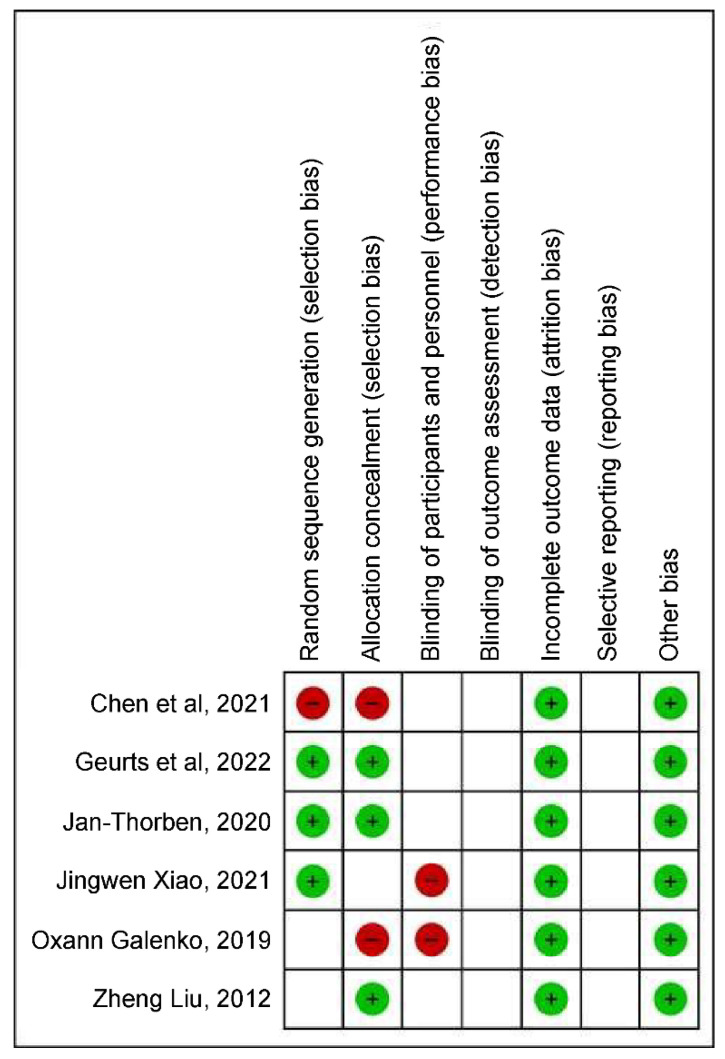
Risk of bias summary: review of authors’ judgments about each risk of bias item for each included study.

The bias assessment indicated a high risk of bias when considering performance and selection biases; however, the types of studies selected should be considered, because each methodology presents different steps. Assuming 100% of the selected studies, a low risk of friction-related bias should be highlighted (Figure 3 and Figure 4).

## 4. Discussion

The present study is the first to use a meta-analysis to evaluate the expression of circulating miRNAs in patients with AF, its effects on AF pathogenesis, and its diagnostic and prognostic implications in AF. AF is a severe health problem whose prevalence increases with age and population survival. It can be classified as paroxysmal or persistent. Clinical manifestations range from palpitations and dyspnea to syncope until it manifests as complications such as embolism or exacerbation of heart failure. Its diagnosis is confirmed by electrocardiography (ECG). Cardiac arrhythmias are monitored by outpatient follow-up and ECG, which are often flawed in the early detection of arrhythmias, especially in asymptomatic patients, and may be associated with poor prognosis [3].

miRNAs have shown significant advantages in their use as biomarkers because they show stable expression after blood sample collection. In addition to being implicated in regulating cellular processes, such as proliferation, differentiation, development, and cell death, they are linked to arrhythmia development processes of the cardiovascular system [16]. Studies by Galenko et al. [17] and Chen et al. [22] reported miRNA-21 as an independent negative predictor of AF that is mainly associated with the development of myocardial fibrosis. This effect is due to the miRNA-21-mediated regulation of the ERK-MAP kinase signaling pathway in cardiac fibroblasts that directly impacts cardiac function and structure by increasing the activity of this kinase, thereby controlling the state of fibrosis and myocardial hypertrophy. The critical role of this miRNA in the development of AF is evident. This highlights the probability of a diagnosis of predisposition and early intervention for AF if the expression of this miRNA is high [19]. miRNA-21 also acts on TGF-β, increasing the fibrotic state [23]. In addition to being directly involved in the pathophysiology of AF, miRNA-21 has been reported in the literature to play a role in the predisposition of other pathologies, such as the complications of type 2 diabetes mellitus. In this situation, a patient with a high expression of miRNA-21 has severe diabetic retinopathy, changes in recurrent tubules due to the propensity to develop interstitial fibrosis, and diabetic cardiomyopathy due to the direct action of the miRNA-21 on fibroblasts, proliferation, and apoptosis of the vascular smooth muscle [24,25].

Among the articles selected in this review, that by Sieweke et al. [18] is the only one that suggested that decreased miRNA-21 levels are associated with the development of AF. Other studies, including that by Barana et al. [26], have highlighted the impact of this miRNA, present in atrial tissue, on the development of AF, through its effect on electrical remodeling. Similarly, plasma miRNA-21 levels were also reported to be associated with myocardial fibrosis.

Galenko et al. [17] showed that low expression levels of miRNA-150 in plasma correlate with AF. Both studies point to miRNA-150 as a regulator of inflammatory mechanisms responsible for the induction of fibrillation and myocardial fibrosis. miRNA-150 has been identified to code for protein genes that act on the pathogenesis of AF, with 18 of the genes found, of which 11 act to regulate the inflammatory response system. These proteins act on the activation of the inflammatory cascade, some related to mechanisms such as apoptosis and others to fibrosis. The relationship between miRNA-150 and the development of AF was also analyzed in a study by Goren et al. [27], in which the effect of AF was observed in patients with heart failure who had low expression of this miRNA. Similar to miRNA-21, miRNA-150 is related to changes in levels of interleukins, such as TGF-β, which are involved mainly in the fibrotic processes—two possible markers of myocardial aggression that can induce AF [28].

Galenko et al. [17] also showed that an increase in the expression of miRNA133a increases the chance of developing AF; however, they did not elucidate the role of this miRNA in the pathophysiology of AF. In a study by Xiao et al. [29], miRNA133a being related to myocardial infarction pointed to this as one of the most abundantly present miRNAs in the heart; in addition, miRNA133a was linked to a series of mechanisms of protection against heart diseases, including cardiogenesis, mediating conductance, automaticity, and potential for cardiac action. miRNA133a also protects against cardiac fibrosis by inhibiting TGF-β and the expression of other factors that promote fibrosis. Thus, it is a biomarker for fibrillation and a potential therapeutic target [29] that could prevent cardiac fibrosis and its complications.

The study by Geurts et al. [19] was the only one to suggest miR-4798-3p and miR-20a-5p as markers of AF. miR-4798-3p presents negative results only among males; however, the mechanisms of interaction of this miRNA with AF are still unknown, and further studies are needed to elucidate these mechanisms. miR-20a-5p has already been reported as a biomarker of other diseases, such as non-small-cell lung cancer, which mainly inhibits neoplastic cell proliferation [11,30]. Therefore, these miRNAs are potential biomarkers, and further studies are required to clarify their roles in the development of AF.

The present meta-analysis was limited by the diversity of the methods of sample collection, quantification, qualification, and miRNA adjustment tools. Moreover, the scarcity of studies that elucidate the mechanism of action of specific miRNAs was a limiting factor in the discussion.

## 5. Conclusions

Using circulating miRNAs as biomarkers is a noninvasive method with high sensitivity and specificity, as shown in our study, and is of great importance in clinical use for identifying AF. Despite the limitations regarding the lack of studies conducted on specific miRNAs and the diversity of methodologies of the studies already elaborated, miRNAs still have great relevance in the diagnosis and early detection of AF.

## Figures and Tables

**Table 1:** Circulating miRNAs and their extraction and quantification methods.

Author	MicroRNA Studied	RNA Extraction Method	MicroRNA Quantification Method	MicroRNA Quantification Technology
Liu [20]	miRNA-150	mirVana™ PARIS™ kit (Invitrogen)	qRT-PCR	TaqMan
Galenko [17]	miRNA-133a	Norgen Biotek, Inc. (Thorold, ON, Canada)	qRT-PCR	TaqMan
Galenko [17]	miRNA-150	Norgen Biotek, Inc.	qRT-PCR	TaqMan
Sieweke [18]	miRNA-21	miRNeasy Mini kit	qRT-PCR	TaqMan
Xiao [21]	hsa-miRNA-4443	miRCURY™ RNA isolation kit	Agilent Bioanalyzer 2100 (Small RNA Analysis Chip; Agilent Technologies, Inc., Santa Clara, CA, USA)	TaqMan
Chen et al. [22]	miRNA-21	TRIzol reagent kit	qRT-PCR	TaqMan
Geurts et al. [19]	miR-20a-5p	Illumina NextSeq 500 (Illumina, San Diego, CA, USA)	HTG Molecular Diagnostics, Tuscon, AZ, USA	------------------
Geurts et al. [19]	miR-4798-3p	Illumina NextSeq 500 (Illumina, San Diego, CA, USA)	HTG Molecular Diagnostics, Tuscon, AZ, USA	-----------------

miRNA: MicroRNA; qRT-PCR: Reverse Transcriptase-Polymerase Chain Reaction; miR: Pre-miRs.

**Table 2:** Description of hypoexpressed and hyperrepressed circulating microRNA in patients with AF.

Author/Year	MicroRNA Studied	Expression in Patients with AF
Galenko [17]	miRNA-133a	Hyperexpressed
Galenko [17]	miRNA-150	Hypoexpressed
Sieweke [18]	miRNA-21	Hypoexpressed
Xiao [21]	hsa-miRNA-4443	Hypoexpressed
Chen et al. [22]	miRNA-21	Hyperexpressed
Geurts et al. [19]	miR-20a-5p	Hyperexpressed
Geurts et al. [19]	miR-4798-3p	Hyperexpressed

AF: Atrial Fibrillation; miRNA: MicroRNA.

**Table 3:** Tabulation of data selected for meta-analysis after the systematic review.

Author/Year	Effect	Result	IC Inferior	IC Superior	Subgroup
Galenko [17]	OR	0.94	0.90	0.98	miRNA-21
Sieweke [18]	RR	0.16	0.04	0.7	miRNA-21
Chen et al. [22]	OR	1.26	1.15	1.37	miRNA-21
Galenko [17]	OR	0.98	0.96	0.99	miRNA-150
Liu [20]	OR	1.96	1.50	3.57	miRNA-150
Geurts et al., 2021 [19]	OR	1.30	0.68	2.58	miR-20a-5p
Geurts et al., 2022	OR	0.64	0.44	0.97	miR-4798-3p
Xiao [21]	ROC	0.84	0.77	0.87	hsa-miRNA-4443
Galenko [17]	OR	1.02	1.00	1.03	miRNA-133a

OR: Odds Ratio; RR: Risk Ratio; ROC: Receiver Operating Characteristic Curve; CI: Confidence Interval.

## Data Availability

The data that support the findings of this study are available on request from the corresponding author.

## Abbreviations

AF	atrial fibrillation
DGCR8:	DiGeorge syndrome critical region 8
ECG:	electrocardiography
HR	hazard ratio
mRNA	messenger RNA
miRNAs	microRNAs;
OR	odds ratio
pre-miRs	precursor miRs
pri-miRs	primary miRs
qRT-PCR	reverse transcriptase-polymerase chain reaction
TGF-β	transforming growth factor-beta
